# Technology Innovation and Guardrails in Elite Sport: The Future is Now

**DOI:** 10.1007/s40279-023-01913-1

**Published:** 2023-10-03

**Authors:** Fergus Guppy, Borja Muniz-Pardos, Konstantinos Angeloudis, Gerasimos V. Grivas, Asimina Pitsiladis, Ross Bundy, Irina Zelenkova, Kumpei Tanisawa, Hiroshi Akiyama, Iphigenia Keramitsoglou, Mike Miller, Melanie Knopp, Fabian Schweizer, Tobias Luckfiel, Daniel Ruiz, Sebastien Racinais, Yannis Pitsiladis

**Affiliations:** 1 Institute of Life and Earth Sciences, School of Energy, Geoscience, Infrastructure and Society, Heriot-Watt University, Edinburgh, UK; 2 GENUD (Growth, Exercise, Nutrition and Development) Research Group, Faculty of Health and Sport Sciences, University of Zaragoza, Saragossa, Spain; 3 Physical Education and Sports, Division of Humanities and Political Sciences, Hellenic Naval Academy, Piraeus, Athens Greece; 4 Human Telemetrics, London, UK; 5 Faculty of Sport Sciences, Waseda University, Tokorozawa, Japan; 6 Graduate School of Sport Sciences, Waseda University, Tokorozawa, Japan; 7 National Observatory of Athens, Athens, Greece; 8 World Olympians Association, Lausanne, Switzerland; 9 adidas Innovation, adidas AG, Herzogenaurach, Germany; 10 Environmental Stress Unit, CREPS Montpellier - Font Romeu, Montpellier, France; 11 Department of Sport, Physical Education and Health, Hong Kong Baptist University, Hong Kong SAR, Hong Kong

## Abstract

A growing number of companies are developing or using wearable sensor technologies that can monitor, analyse and transmit data from humans in real time that can be used by the sporting, biomedical and media industries. To explore this phenomenon, we describe and review two high-profile sporting events where innovations in wearable technologies were trialled: the Tokyo 2020 Summer Olympic Games (Tokyo 2020, Japan) and the 2022 adidas Road to Records (Germany). These two major sporting events were the first time academic and industry partners came together to implement real-time wearable solutions during major competition, to protect the health of athletes competing in hot and humid environments, as well as to better understand how these metrics can be used moving forwards. Despite the undoubted benefits of such wearables, there are well-founded concerns regarding their use including: (1) limited evidence quantifying the potential beneficial effects of analysing specific parameters, (2) the quality of hardware and provided data, (3) information overload, (4) data security and (5) exaggerated marketing claims. Employment and sporting rules and regulations also need to evolve to facilitate the use of wearable devices. There is also the potential to obtain real-time data that will oblige medical personnel to make crucial decisions around whether their athletes should continue competing or withdraw for health reasons. To protect athletes, the urgent need is to overcome these ethical/data protection concerns and develop wearable technologies that are backed by quality science. The fields of sport and exercise science and medicine provide an excellent platform to understand the impact of wearable sensors on performance, wellness, health, and disease.

**Supplementary Information:**

The online version contains supplementary material available at 10.1007/s40279-023-01913-1.

## Key Points

**Table:** 

The integration of accurate wearable sensors and real-time transmission of data represents a unique opportunity to protect the health of athletes during training and competition, as demonstrated in the pilot implementations during the Tokyo 2020 Olympics and the 2022 adidas Road to Records event.
The monitoring of meteorological conditions [prioritising wet bulb globe temperature (WBGT) monitoring] in situ at the athletes’ specific location during competition, offers valuable and precise information to protect athletes from exertional heat illnesses.
Despite the undoubted benefits of real-time metrics and wearables, there are well-founded concerns such as insufficient validity testing, data privacy issues, information overload and exaggerated marketing claims.

## Introduction

The incorporation of recent technology that aims to enhance performance, athlete safety or the overall fan experience has become a vital part of elite sport. Examples include the use of video assistant referees in football [1], advanced analysis of pitcher performance on return from injury in baseball [2] and foot-worn inertial sensors providing better understanding of running economy during distance running [3]. The rapid development of these technologies in the context of elite sport means that the guardrails associated with the rules and regulations put in place to ensure safety, fair play and the integrity of competition must be updated based on science to prevent any one team or individual from gaining an unfair advantage and to maintain a level playing field.

A variety of wearable sensor technologies worn on, close to, or even in the body that can monitor, analyse, transmit and/or receive data from other devices and/or cloud services to provide feedback in real time to the user are being developed [3–8]. An ever-increasing number of companies developing wearables are receiving considerable attention from the sporting and biomedical community, including those in broadcasting and on social media. Employment and sporting rules and regulations need to evolve to facilitate the use of wearable devices [9, 10]. Here we review two major sporting events where important innovations in wearable technologies are being carefully and gradually introduced such as the real-time monitoring of biometrics at the Tokyo 2020 Summer Olympic Games (Tokyo 2020, Japan) and adidas Road to Records (Germany). These two high-profile sporting events represent the first concerted efforts involving academic and industry partners to systematically implement real-time wearable solutions during elite competition. Other pertinent recent examples will also be introduced such as the non-invasive, in situ monitoring of sweating rate and sweat electrolyte losses, and augmented reality contact lenses used as a display that allow the athlete to be informed on best performance strategies.

Despite the undoubted benefits of wearables, there are well-founded concerns regarding their implementation including: (1) limited evidence quantifying the potential beneficial effects of analysing specific parameters, (2) the quality of hardware and provided data, (3) information overload, (4) data security and (5) exaggerated marketing claims [11–13]. These concerns will be highlighted with particular focus on the two major sporting events reviewed, and by highlighting these concerns, we suggest that wearable devices that use biological data for sport performance undergo rigorous evaluation ensuring that these devices have received a certification of veracity [1, 2] or guiding reference [14] prior to acceptance of use in elite sporting competition. This quality control process would undoubtedly be highly sought after by those individuals using them for general health monitoring.

The two sporting events we review here will also help highlight some of the potential ethical considerations for governing bodies, sports organisers, the labour market/industry and the medical field in general [7, 15, 16], with, for example, many unresolved issues relating to data protection such as ownership and confidentiality. Nevertheless, these concerns should not be used to block the use of such technology. Instead, systematic efforts should be adopted to write the ‘playbook’ on how best to integrate this technology for the benefit of the athlete, athlete support staff, the event organisers including international federations, broadcasters and medical personnel responsible to protect the health of the athletes, spectators, and officials. For example, the implementation of this technology and the obtaining of real-time data will also oblige medical teams to make crucial decisions around their athletes continuing competing or withdrawing from their event. Much more development is needed both technologically and in terms of rules of the sport before such solutions can be fully adopted for the safety of athletes.

As will become clear from the present review, a key priority for all those stakeholders involved in technological support of elite athletes and sporting events, is to overcome important ethical/data protection concerns and develop wearable technologies that are backed by quality science. New sponsors and major investment will be required to facilitate this progress. The field of sport and exercise science and medicine provides an excellent platform to understand the impact of wearable sensors on performance, wellness, health and disease [17]. Through sport, individualised prescription, performance enhancement and protection of the health of users can be transferred from elite athletes to the recreational athlete, the wellness industry, patients and emergency services. The aim of this manuscript is to inform on the implementation process of the real-time monitoring technology in elite sport, presenting some of the early data collected during two relevant high profile sporting events: first at Tokyo 2020 and second at the 2022 adidas Road to Records. Additionally, we aim to provide a guide for athletes, coaches, engineers/industry and administrators for subsequent technological applications during major sporting events.

## Tokyo 2020

A major recent innovation in wearable technologies was the application of real-time monitoring at Tokyo 2020, consisting of a smartwatch application and ecosystem designed to collect, process and transmit a wide range of physiological, biomechanical, bioenergetic and environmental data using cloud-based services [3]. This represented the first concerted effort involving academic and industry partners to systematically implement real-time wearable solutions to protect the health of athletes competing in major sporting events conducted in hot and humid environments such as were seen at the Olympic Games in Tokyo, and transmitting the athlete’s bioenergetic response in real time. The idea to implement wearable technologies and real-time monitoring of different athletes and sports emerged from discussions by members of the *Adverse Weather Impact Expert Working Group* created by the International Olympic Committee (IOC) to proactively protect the health of athletes competing in Tokyo 2020 given that the environmental conditions in Tokyo were predicted to be extreme [16, 18].

The *Adverse Weather Impact Expert Working Group* of the IOC instigated numerous developments to help protect the health of athletes competing in the heat in Tokyo 2020 and beyond to Paris 2024. One such development was building on the success of the Doha 2019 World Athletics Championships assessment of core body temperature and the impact of different cooling strategies on heat distribution measured via thermal cameras and athletic performance [19] to develop live-transmitting technology that allows the tracking of multi-source data within a single application. Specifically, the developed ecosystem provides live feedback of core temperature, heart rate and a range of biomechanical variables facilitated through a cloud-based portal allowing the athlete support team to view the data in real time anywhere with internet or mobile access [4, 5]. This technology has the potential to help in the management of athletes during a medical emergency to instantly orient the diagnosis and accelerate a potential intervention. Combining core and skin temperature responses that are associated with collapse and/or with withdrawal from competition with biomechanical parameters that can identify disturbances in gait could help in early identification by the medical staff of possible aggravated hyperthermia situations [20].

### Tokyo 2020: *Methods*

A cross-sectional observational and descriptive approach was used to study athletes participating in Tokyo 2020. The approaches, previously described in detail [3, 21], allow the monitoring of biomechanical, physiological and bioenergetic responses of the athletes in real time using the latest wearable technology originally intended for numerous Olympic sports at considerable risk of exertional heat stroke (e.g. 5000 m, 10,000 m, marathon, 20 km race walk, 50 km race walk). However, due to COVID-19 restrictions ranging from limited accreditation of the research group to attend Tokyo 2020 (*n* = 2 researchers) and other restrictions described previously [22] and outlined in the Olympic playbooks [23], only a small number of athletes from a very limited number of Olympic events (i.e. 10,000 m, marathon, 20 km race walk), eventually participated in the study.

#### Athlete recruitment

In the weeks prior to Tokyo 2020, National Olympic Committee team physicians were contacted and their athletes invited to participate in this research aimed at exploring the potential for wearable technology to monitor a range of physiological and biomechanical responses during strenuous exercise/training, including sporting competition, to characterise the thermoregulatory response of the athletes participating in different events and to help in the early identification of heat illness during competition in extremely hot environments such as Tokyo 2020 and Paris 2024. Interest to participate in this research was declared prior to the games; no athletes/coaches were recruited during the games, in line with the approval obtained from the IOC.

#### Testing at the Olympic Games

Due to COVID-19 restrictions and in accordance with the Olympic playbooks [23], there was minimal to no contact with the athletes at the games. This necessitated the development of extremely user-friendly technology. For example, the operation of the smartwatch and application was restricted to the pressing of three buttons in simple succession. Athletes were provided with all the technology needed soon after their arrival in Japan to allow sufficient time for familiarisation. Originally, the plan was for the technology to be provided to the athletes in the country of origin so they could familiarise themselves with the technology well in advance. Unfortunately, this was not possible due to COVID-19 restrictions [22].

**Fig. 1:**
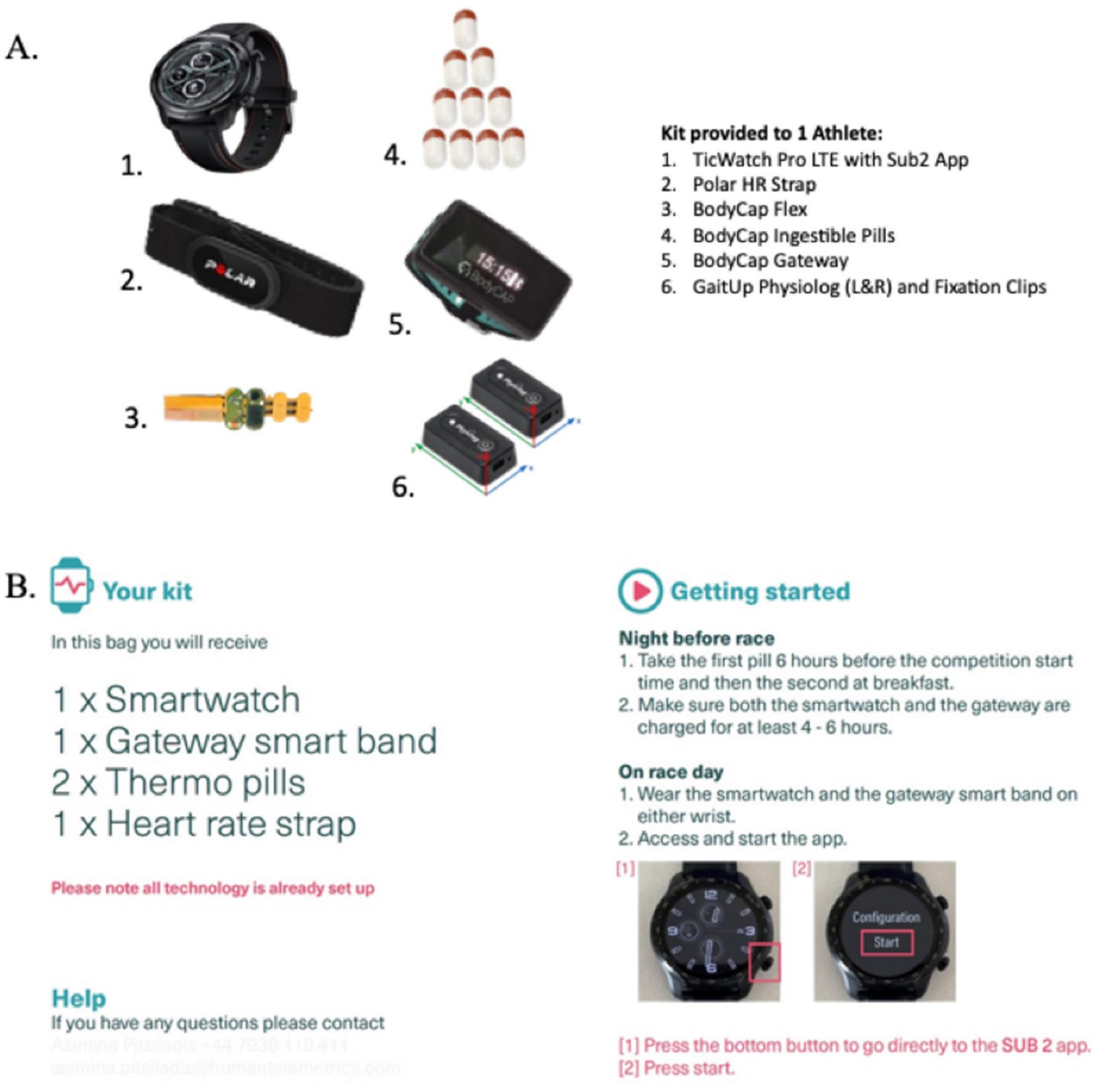
The technology available to each athlete (**a**), and the instructions provided to athletes prior to the event (**b**)

All athletes participating in the study agreed to have their core body temperature measured as the primary outcome. Athletes had the option to have other metrics assessed during training and/or competition from a list of available sensors. Athletes also had the option to personalise the setup of the smartwatch to only show the data of interest (e.g. core body temperature or heart rate) during training and/or competition. Athletes further could choose real-time transmission of their data or offline recording, with real-time monitoring achieved by a small wrist worn bracelet (Gateway smart band; Fig. 1a) that, alongside a smart watch (TicWatch Pro3 Cellular/LTE, Mobvoi, Beijing, China) and bespoke Sub2 application (Human Telemetrics, London, UK), transmitted the information wirelessly through cellular connectivity to the research team via the Cloud. Cellular connectivity in Japan was achieved using e-SIMs activated either in Spain or the UK (Vodafone, Spain, and UK) and the smart watch set in roaming. For some unknown reason, cellular connectivity in roaming mode did not function well initially while in Japan, resulting in repeat crashing of the smartwatch. This problem remained throughout the first week of the games, until this issue was resolved following extensive troubleshooting by simply setting the smartwatch to 3G rather than 4G. Data could be downloaded from the sensors at the end of the event without the use of live transmission of the data; however, no athlete chose this option.

For core body temperature assessment, athletes were required to swallow the temperature pill (eCelsius, BodyCap, Caen, France) 6 h prior to their event and in line with the advice from manufacturers. To ensure athletes, coaches and medical staff were well familiarised with the temperature pills, unused examples were made available to athletes in the weeks prior to the event.

#### Optional measurements

Athletes agreeing to participate in this research had consented, in addition to the measurement of core body temperature, to at least one of the following sensors to be used during training and/or competition. Details of all sensors were made available to participants on request for inspection to alleviate any concerns about size/bulk prior to participating in the study.

*Heart rate*. Athletes had the option to have their heart rate measured using a telemetric heart rate monitor chest strap (H10 Polar Electro Oy, Kempele, Finland) or the inbuilt smart watch sensor (TicWatch Pro3 Cellular/LTE, Mobvoi, Beijing, China). The reason for selecting chest strap-based monitors rather than wrist-worn monitors is based on the greater accuracy of chest strap-based sensors, especially during exercise [24].

*Skin temperature*. Temperature pills (eCelsius, BodyCap, Caen, France) were adapted by the manufacturer to allow skin temperature to be measured. This involved ‘flattening’ of the pill electronics in a manner that ensured the thermistor remained in contact with the skin at all times (i.e. BodyCap flex, Fig. 1a). The skin temperature sensor was positioned with the help of a standard heart rate strap for the measurement of heart rate. This is in line with other manufacturers in the field that also use thermistors in contact with the skin for measurement of skin temperature (e.g. HQ Inc, Florida, USA).

*Stride and foot mechanics sensor*. A foot-worn inertial sensor (Physilog, Gait^up^, CH) that allows 9° of freedom (three-axis accelerometer, gyroscope and magnetometer) and sampling frequency of up to 1000 Hz, with the accelerometer measurement ranging up to ± 16 g, was placed on each shoe to measure spatiotemporal components of the lower extremities, such as the contact time (s) and strike angles (degree) of each foot, cadence (steps/min), and foot mechanics variability. The sensor was inserted in a rubber case provided by the manufacturer and attached to the laces of the athletes’ shoe. Raw data from the Gait^up^ foot sensor were collected following a calibration process conducted in accordance with the manufacturer’s recommendations. Calibration involved establishing the relation between sensor output and physical quantities such as acceleration or angular velocity. Data from multiple sensors, such as accelerometers, gyroscopes and magnetometers, were fused together by the particular sensor’s default setting to obtain more accurate measurements, reducing noise in the data. For further removal of noise and artefacts from the raw data, semi-automatic digital signal processing and filtering methods were applied post-event via Gait^up^ proprietary software (PhysiRunLab; Physilog RTK, Gait^up^, CH). The foot-worn inertial sensors can transmit data over Bluetooth low-energy connectivity, allowing for real-time data streaming. The abovementioned variables were selected to examine the variability of ground contact time as a potential indicator of heat stroke or injury, as well as the sustained wide change of contact time during the race as a marker of fatigue. Given also that the cadence and strike angles show the runners’ commitment, as well as performance strategies (i.e. acceleration, deceleration, steady-state pace, etc.) and techniques (i.e. forefoot strikes, midfoot strikes, etc.), respectively, these variables and the variability within these factors during the race may indicate fatigue, injury or heat stroke [25].

#### Environmental Conditions

For monitoring ambient conditions, wet bulb globe temperature (WBGT) is typically recommended and used, requiring a true measure of natural wet bulb temperature, a dry-bulb thermometer shaded by a structure eliminating effects of thermal radiation and with mechanically aspirated ambient air and a black globe with a diameter of 15 cm. Although most portable devices do not match those specifications, for example, no aspirated ambient air temperature, the Kestrel (5400 Heat Stress Tracker, Boothwyn, Pennsylvania, USA) is considered a reasonable compromise [26], acceptable for use in field monitoring studies [27] and calibrated with a reported maximum relative expanded uncertainty of ± 0.4 °C for temperature, ± 1% for relative humidity, ± 0.3 hPa for barometric pressure and ± 1% for wind speed within the airspeed range 3.6–19.9 m/s and ± 1.7% within the airspeed range 0.9–3.6 m/s (Kestrel 5400 Heat Stress Tracker Certificate of Conformity, Boothwyn, Pennsylvania, USA).

Ambient temperature was monitored and recorded using the Kestrel devices (Kestrel 4400, Nielsen-Kellerman, Boothwyn, USA) at all venues of the Olympic Games where data collection took place. The Kestrel devices were linked by Bluetooth to the real-time monitoring system via a bespoke cellular wireless transmitter (Human Telemetrics, London, UK) that allowed the ambient conditions generated by the Kestrel to be displayed in real time on a dashboard to the research team via the Cloud.

The application developed for Tokyo 2020 also provided a live data feed of air and land surface temperature together with relative humidity [20]. Collecting air temperature and relative humidity data from static weather stations may fail to reflect the spatial variations of these variables due to the sparsity of the network. A unique development briefly described elsewhere [3] has involved the tracking of the actual heat experience of the individual (i.e. the SCOUTS model) [28]. The SCOUTS model was designed to minimise heat stress in individuals and urban communities by using ‘Mobile Crowdsensing’, which allows the model to gather data at much finer spatial–temporal granularities compared with traditional methods. In addition, a complementary solution involves downscaling weather forecast data with satellite data at the athlete’s location using advanced machine learning algorithms. This innovation is especially important in regions where weather station networks are absent. This digital approach permits seamless transition to any global location, provision of ambient conditions for each athlete and endless possibilities to scale up to include more parameters such as forecast of upcoming ambient conditions, UV index and air quality indices. Our technological solution (www.extrema-global.com provided by ARTi Analytics BV​; Rotterdam, the Netherlands) integrates real-time data transmission including ambient conditions from downscaled modelled data via an Application Programming Interface connection in pre-designated areas such as the Tokyo prefecture, including Sapporo where the marathon and race-walking events took place. The user/athlete makes use of the digital infrastructure to have the required information readily available; see Table 1 for ambient conditions during three Olympic events (measured = Kestrel, and derived = extrema) at Tokyo 2020.

**Table 1:** Heart rate (device and strap), core body temperature and ambient conditions (measured = Kestrel, and derived = extrema) at Tokyo 2020

Metric	10,000 m	20 km Race walk	Marathon
Heart rate device (bpm)			
Average	186	178	162
Maximum	200	211	190
Heart rate strap (bpm)			
Average	n/a	168	n/a
Maximum	n/a	178	n/a
Core body temperature (°C)			
Average	39.5	39.2/39.2^a^	38.6
Maximum	40.2	39.6/39.8^a^	38.8
Ambient conditions (measured)			
Temperature (°C)			
Start	28.3	32.2	27.3
End	26.1	34.5	27.7
Relative humidity (%)			
Start	77.3	99.0	73.5
End	79.1	63.2	78.7
Wind speed (m/s)			
Start	0	0	0.4
End	0	1.0	0.5
WBGT (°C)			
Start	26.1	33.7	25.2
End	26.1	32.8	25.9
Ambient conditions (derived)			
Temperature (°C)			
Start	27	28	24
End	28	26	26
Relative humidity (%)			
Start	70	65	81
End	69	80	76

^a^Athlete used a two-pill strategy

Data collection occurred at numerous Olympic venues across the Tokyo Games. Only official event locations were used, with researchers requiring accreditation from Tokyo 2020 to access these venues, and testing taking place between 24 July and 9 August 2021.

### Tokyo 2020: *Results*

*Windsurfing Practice Session (Enoshima Island, Sagami Bay, Kanagawa Prefecture, Japan)*. Windsurfers were unable to use our technology in competition in Tokyo 2020 due to Global Positioning System (GPS)-enabled tracking devices that could aid navigation and decision-making being prohibited during competition, although this prohibition was not explicitly stated in the competition rules [29] but specified in the notice of race (NoR) and/or sailing instructions (SIs). We were able to test the technology during a practice session prior to the competition. This practice session revealed the unexpected instability problem with cellular connectivity unknown to the research team due to the lack of testing of our technology in Tokyo prior to the start of the Olympic Games due to COVID-19 travel restrictions.

**Fig. 2:**
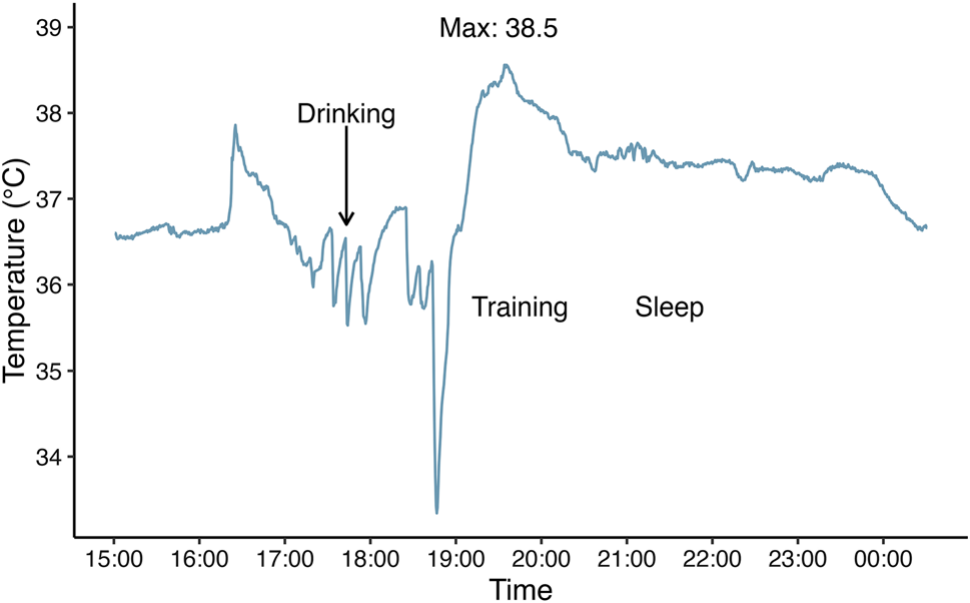
Core body temperature data of an athlete the day prior to the Olympic 10,000 m men’s final in Tokyo 2020

*Testing in the Olympic Village (Tokyo, Japan).* Having resolved the difficulties with cellular connectivity, we were able to instrument an athlete the day prior to the 10,000 m men’s final to test our technology and allow the athlete time to familiarise himself with the technology. The technology worked well and revealed some clear patterns in the life of the athlete with periods of fluid ingestion, training and sleeping clearly visible in changes in core temperature (Fig. 2).

*10,000 m Men’s Olympic final (Tokyo, Japan).* An athlete in the 10,000 m Olympic final consented to use the technology during the race but opted not to wear the heart rate monitor with connected skin temperature or the foot sensors. Considering this and following-up the principle of not overloading the athletes with the proposed full technological package when it was not necessary, a proprietary ‘physilog’ watch-based running algorithm (Physiolog5, Gait^up^, CH) was developed and used. Therefore, this developed algorithm allowed the collection of spatial–temporal variables such as ground contact time, cadence and running vertical oscillation, via a running biomechanics database integrated into the watch, without the need to use the foot sensors.

Due to COVID-19 restrictions, we were unable to contact the athlete, hence instructions were provided remotely (Fig. 1b). These instructions included the procedures to keep the battery of the devices charged for as long as possible prior to training or competition and how to connect the sensors to the smart watch. Instructions were also provided to athletes in video format to warrant its understanding; see Table 1 for heart rate, core body temperature, and ambient conditions (measured and derived) measured at this Olympic final. The data revealed a high average heart rate (186 bpm) and a very high maximum heart rate (200 bpm) that may reflect the use of the sensor incorporated in the smart watch (i.e. the athlete did not wish to use the heart rate strap). Average core body temperature was high (39.5 °C) with a maximum core body temperature of 40.2 °C reflecting the high-intensity effort, characteristic of a 10,000 m final being run during hot and humid conditions as measured and derived (Table 1).

**Fig. 3:**
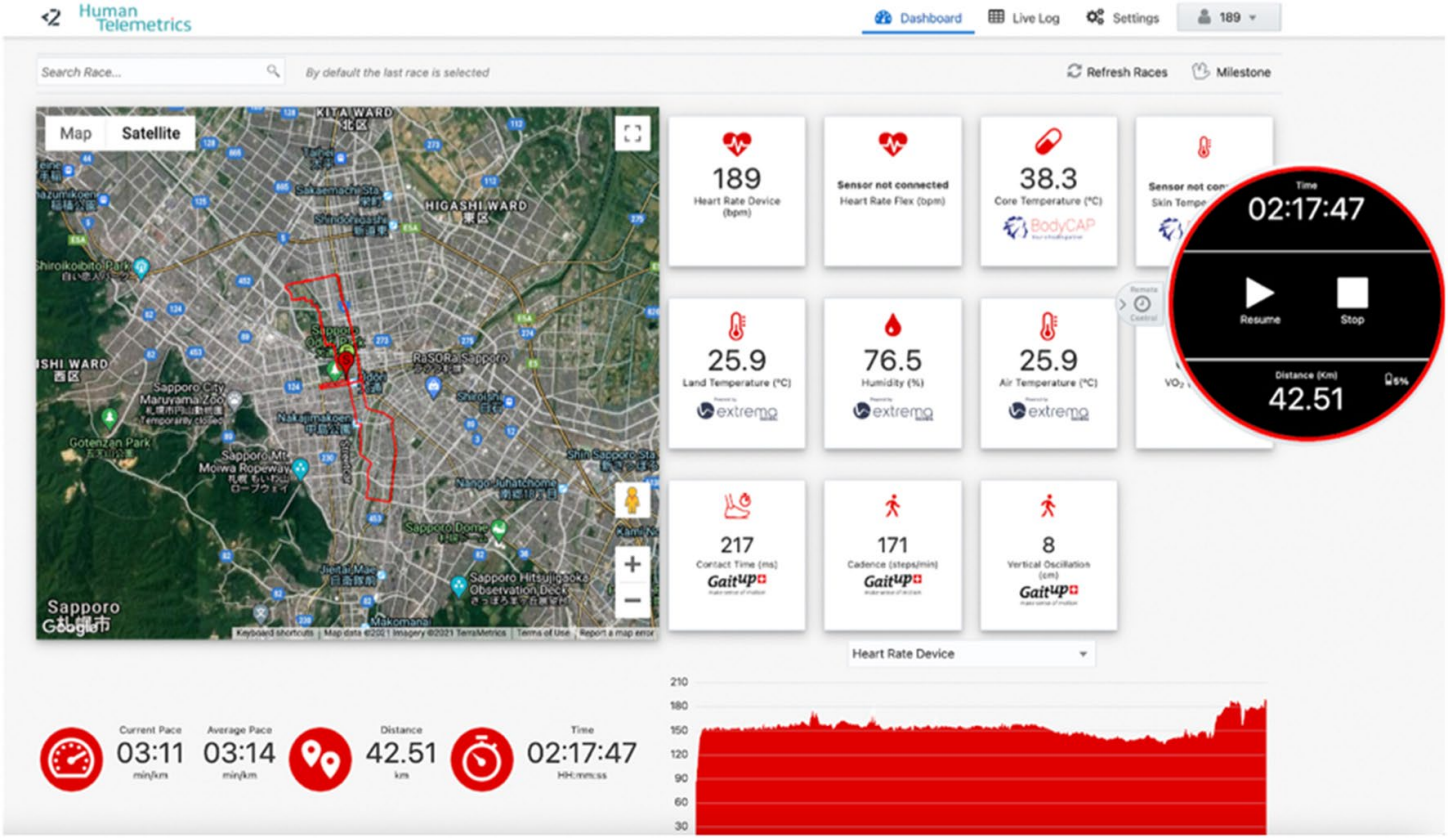
Screenshot of the athlete’s metrics in the men’s marathon Olympic final at Tokyo 2020

*Men’s Marathon Olympic final (Sapporo, Japan).* The athlete involved in the 10,000 m final found the technology helpful and unobtrusive and volunteered to use the technology again during the Olympic marathon that was held in Sapporo some days later, with heart rate, core body temperature and ambient conditions (measured and derived) measured at this Olympic final (Table 1, Fig. 3). Note the lower average heart rate (162 bpm) and lower maximum heart rate (190 bpm) achieved by this athlete during the marathon, run during similar ambient conditions as those the same athlete experienced during the 10,000 m final a few days earlier in Tokyo (Table 1). This may also explain the approximately 1.4 °C lower average and maximum core body temperature measured in this athlete during the marathon versus the 10,000 m final (Table 1).

*Men’s 20 km Race Walk Olympic final (Sapporo, Japan).* A race walker volunteered to use the technology after trialling the technology during training for a week. This athlete also found the technology helpful and unobtrusive. Heart rate, core body temperature and ambient conditions (measured and derived) were measured at this Olympic final (Table 1). Note the substantially higher average (178 bpm versus 168 bpm) and maximum (211 bpm versus 178 bpm) heart rate using the smart watch device versus heart rate strap, respectively. This difference of approximately 10 bpm for average heart rate and 33 bpm for maximum heart rate most likely reflects measurement error often associated with the use of the sensor technology incorporated in the smart watch.

This athlete used a two-temperature pill strategy, having taken one temperature pill in the morning and the other in the afternoon before the afternoon race (16:30 on the 5 August 2021). This timing difference did not seem to influence the measurement of core body temperature, and the similarly high average and maximum core body temperature measurements between temperature pills would suggest that both pills had passed the stomach and, therefore, the measurements were not affected by the athlete's drinking strategy as opposed to the training session illustrated in Fig. 2. The similarly high core body temperature in this race walker compared with the athlete running in the 10,000 m, and substantially higher in the race walker by 0.6–1.0 °C compared with the athlete in the marathon, is probably a reflection of the hotter conditions during the race walk (i.e. WBGT 7–9 °C higher during the race walk compared with the 10,000 m and marathon; Table 1).

*Other athletes:* Another male athlete volunteered to use the technology in the Olympic marathon. The athlete was instrumented but failed to open the Sub2 application or closed it in error; consequently we were unable to collect or transmit his performance metrics. Three female race walkers involved in the 50 km race walk and another female race walker involved in the 20 km race walk initially volunteered to use the technology during competition at the Games but withdrew in the days before their race mainly due to COVID-19 restrictions. There was also interest in using the technology in the Olympic Equestrian events, but again COVID-19 restrictions made its implementation impossible. An additional interest also emerged to use the technology in the 10,000 m Olympic open water swim in both men’s and women’s races but the technology in Tokyo at the time did not allow the smart band to function when positioned around the ankle as requested by the swimmers due to the increased distance from the Gateway smart band to the pill in the gastrointestinal tract.

### Tokyo 2020: *Discussion and Concluding Remarks*

The integrative solution described here and elsewhere [3, 4] represents the first real-time, integrated and remote system that can monitor and analyse both health- and performance-related information, obtaining data from the body and the environment and providing instantaneous feedback to the athlete/coach/scientist/broadcaster. The measurement of accurate environmental conditions includes the use of WBGT monitoring combined with the use of portable weather stations at the competition sites, while accurate core temperature monitoring requires the implementation of ingestible telemetric pills. Additional wearable sensors such as inertial measurement units or sweat/electrolyte patches may be incorporated, with all these technologies integrated within the same ecosystem and able to transmit in real time with less than a 1 s delay. Notably, despite the efforts of our team on the ground, COVID-19 restrictions only allowed us to test a small group of athletes from just a few sporting disciplines, with little chance for a proper familiarisation with the technology, which was therefore a limitation during the recruitment process. Following its pioneering implementation at Tokyo 2020 and as the COVID-19 restrictions were relaxed, we proceeded to furtherly develop and refine this real-time technology to serve as a ‘hub’ to aggregate a much larger range of data feeds to protect the health of athletes, help characterise and understand performance at an individual level, as well as to enhance the broadcast of sporting events with the relay of interesting performance-metrics and biometrics to the spectator.

## adidas Road to Records

Road To Records is a special day of elite racing organised by adidas, the sportswear manufacturer, and held at adidas HQ (Herzogenaurach, Bavaria) with the express aim of breaking records in the 5 km, 10 km and half marathon. The events are open to the public and often have a charitable component, with proceeds going towards a chosen cause. The goal of the series is to provide an opportunity for people of all abilities to challenge themselves, set personal records, and be a part of a community of runners. As part of the 2022 adidas Road to Records event, our team was invited to incorporate our wearable technology ecosystem to monitor, in real time, the performance of elite athletes in the 5 km, 10 km and half marathon events. One major development in wearable technology includes advances in devices that measure running biomechanics with their efficacy demonstrated during a variety of different conditions [5, 30, 31]; these technologies are already being used to analyse gait in athletic [32] and clinical populations [33]. The interest in these, especially in running, has led to the development of specific algorithms that examine individual athlete's running mechanics [5, 30, 34], with additional benefits of real-time data collection facilitating feedback to athletes and their support teams [5].

Understanding foot mechanics is crucial in endurance athletes, as shown by the recent surge in world record performances since the inclusion of advanced footwear technology. There are numerous factors such as foot strike patterns [35], lower ground reaction forces [36], shorter ground contact times [37], greater stride angles [38], lower cadence and longer strides [39], all of which lead to better running kinematics and, in consequence, a reduced energy cost of running. However, the assessment of running biomechanics is traditionally achieved in the laboratory with its intrinsic limitations, such as bouncy treadmills altering biomechanical and bioenergetic responses of athletes [40], and as such, being able to monitor athlete performance during a ‘real’ event has been limited. In particular, instrumented force-sensing treadmills, motion capture systems and other novel technologies (e.g. force- and pressure-sensing walkways, infrared electronic mat technology, etc.) that can capture kinematic and kinetic responses have been widely used during laboratory-based biomechanical experiments. While laboratory assessments are useful for studying running biomechanics in controlled conditions, they may not accurately reflect an athlete’s performance in real-world situations. However, advancements in wearable technology and portable sensors have allowed for more accurate assessments of running biomechanics in the field [41]. It is important to continue exploring and utilising these technologies to improve our understanding of athlete performance. As a result, a major aim of this event was to monitor, in real time, the running mechanics of elite athletes taking part in the 5 km, 10 km and half marathon events, while further establishing our wearable technology monitoring ecosystem that has been utilised in other events such as the 2022 Brighton Marathon, as well as at the Tokyo 2020 Olympic Games as an important tool in the future use of telemedicine approaches in elite sport and beyond.

### adidas Road to Records: *Methods*

Twenty-six athletes taking part in the Road to Records event agreed to wearing additional sensors during their event, with these athletes split between the 5 km (*n* = 10), 10 km (*n* = 7) and 21 km (*n* = 9) events. The mean performances of these athletes in their relevant events are presented in Table 2.

**Table 2:** Performance data of the athletes included in our study from adidas Roads to Records 2022

Event	Time (s)	Time (min:s)
Female 5 km (*n* = 5)	930 ± 17	15:30 ± 00:17
Male 5 km (*n* = 5)	796 ± 19	13:16 ± 00:19
Female 10 km (*n* = 4)	1923 ± 49	32:02 ± 00:49
Male 10 km (*n* = 3)	1692 ± 49	28:12 ± 00:49
Female 21.1 km (*n* = 5)	4063 ± 20	59:49 ± 00:20
Male 21.1 km (*n* = 4)	2589 ± 19	67:43 ± 00:19

Data shown are mean ± standard deviation

#### Outcomes

During individual events, participants wore a foot-worn inertial sensor present in the shoelaces of their chosen shoe (ORPHE CORE 3.0 sensor, Orphe Inc., JP). This sensor is equipped with a nine-axis motion sensor, which includes a three-axis accelerometer, a three-axis gyroscope and a three-axis magnetometer with a sampling frequency of up to 200 Hz. The ORPHE CORE 3.0 IMUs have a dynamic range of ± 16 g for acceleration measurements, allowing accurate measurement of accelerations associated with physical activities with high accelerations such as sprinting, without the risk of signal saturation or clipping. It also uses Bluetooth low energy for wireless communication with other electronic devices. The ORPHE CORE 3.0 sensor was used to measure flight time, cadence and stride length for both the left and right leg, as well as a heart rate monitor for the recording of real-time heart rate. In addition, satellite-based GPS data were used to map these data to the athlete’s position on the track. These data were collated utilising the smart watch ecosystem further explained elsewhere, and comprised the same technology that was successfully implemented at the Tokyo 2020 Olympic Games [3, 5]. Briefly, this technology links a smart watch with an e-SIM and connects to the inertial sensor using Bluetooth with these data transmitted to a central data server allowing for real-time monitoring of performance metrics as well as for storage for later offline analysis.

#### Data Analysis

Due to the novel and explorative nature of these data, as well as the relatively small sample of elite athletes, no statistical analysis was conducted on this data set, with quantitative data presented as individual responses. All analysis and data processing was conducted using the statistical programming language R (RStudio, PBC, Boston, MA) utilising the tidyverse group of packages [42] to visualise these data and remove any outliers more than 1.96 standard deviations from the originally calculated mean. We appreciate that applying statistical models to this data set is not appropriate and it is not our intention to apply these findings to populations of athletes, with further work required before that is possible.

### adidas Road to Records: *Results*

Spotting spurious patterns in data is a natural part of human learning [43], and as a result, we are deliberately not reporting large quantities of data here to avoid over interpreting outcomes in a small sample of athletes. However, we want to showcase the data that can be generated with the approach we have adopted here to allow researchers, support staff and athletes to assess the utility of incorporating this methodical approach.

**Fig. 4:**
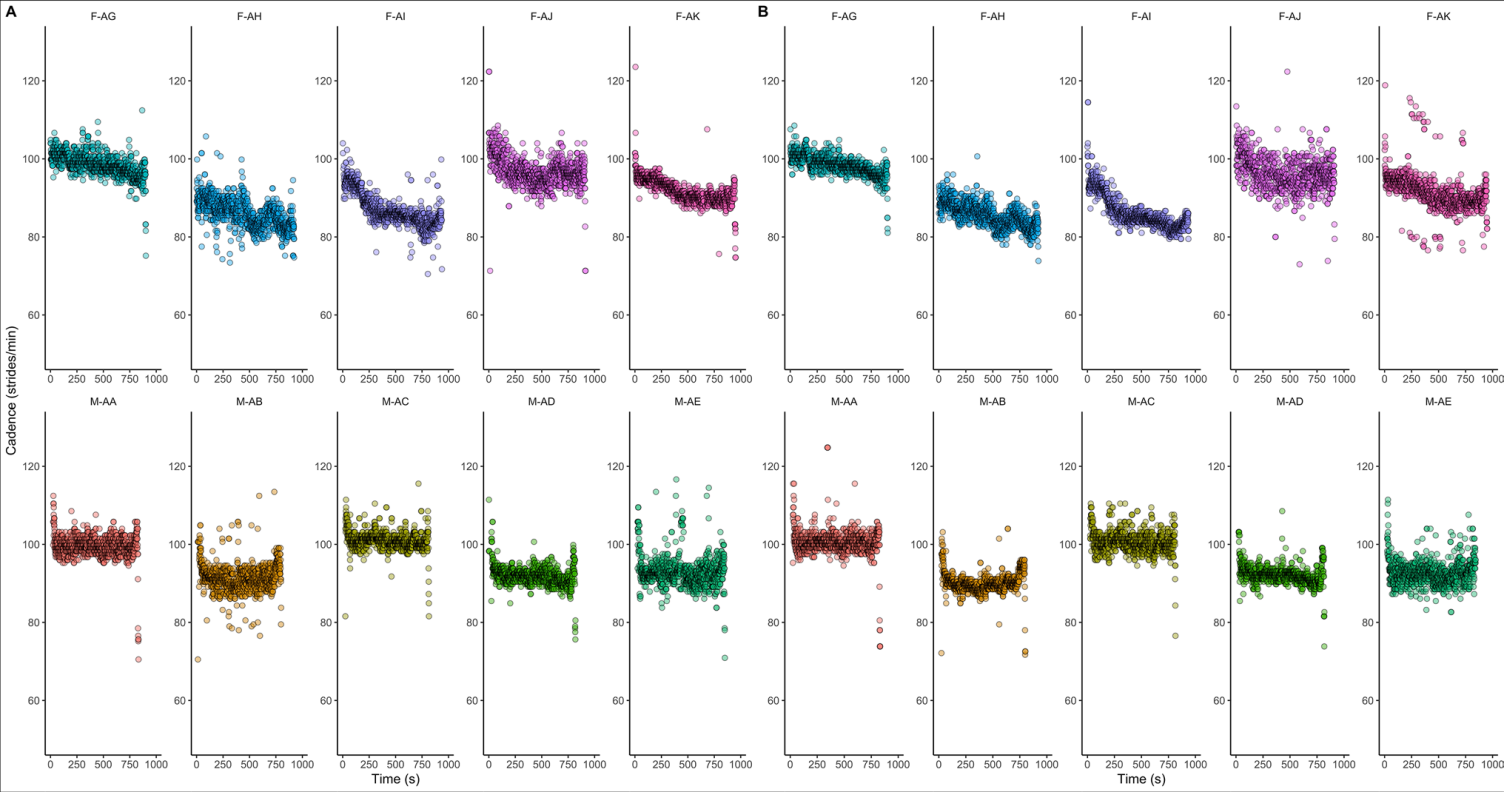
Cadence (strides/second) of left foot (**a**) and right foot (**b**) during the 5 km event at the 2022 adidas Road to Records event. F: female participants; M: male participants

*Cadence.* The cadence of individual athletes during the 5 km event varied from 1.42 ± 0.05 to 1.64 ± 0.04 strides/s in the right foot and from 1.43 ± 0.09 to 1.64 ± 0.05 strides/s in the left, with the individual responses for each athlete during the 5 km available in Fig. 4. Female athletes showed slower mean cadence (1.54 ± 0.11 and 1.56 ± 0.10 strides/s in the right and left foot, respectively) compared with males (1.56 ± 0.09 and 1.57 ± 0.10 strides/s in right and left foot, respectively).

During the 10 km race, cadence varied from 1.52 ± 0.06 to 1.65 ± 0.06 strides/s in the right foot and from 1.54 ± 0.18 to 1.64 ± 0.07 strides/s in the left, with the female athletes showing slower mean cadence (1.58 ± 0.10 and 1.58 ± 0.08 strides/s in right and left foot) compared with males (1.62 ± 0.07 and 1.62 ± 0.07 strides/s in the right and left foot, respectively). Finally, during the 21.1 km event, cadence varied from 1.45 ± 0.03 to 1.55 ± 0.06 in the right foot and from 1.46 ± 0.03 to 1.53 ± 0.08 in the left, with the female athletes showing slower mean cadence (1.50 ± 0.09 and 1.51 ± 0.11 in the right and left foot, respectively) compared with males (1.52 ± 0.10 and 1.52 ± 0.07, respectively).

**Fig. 5:**
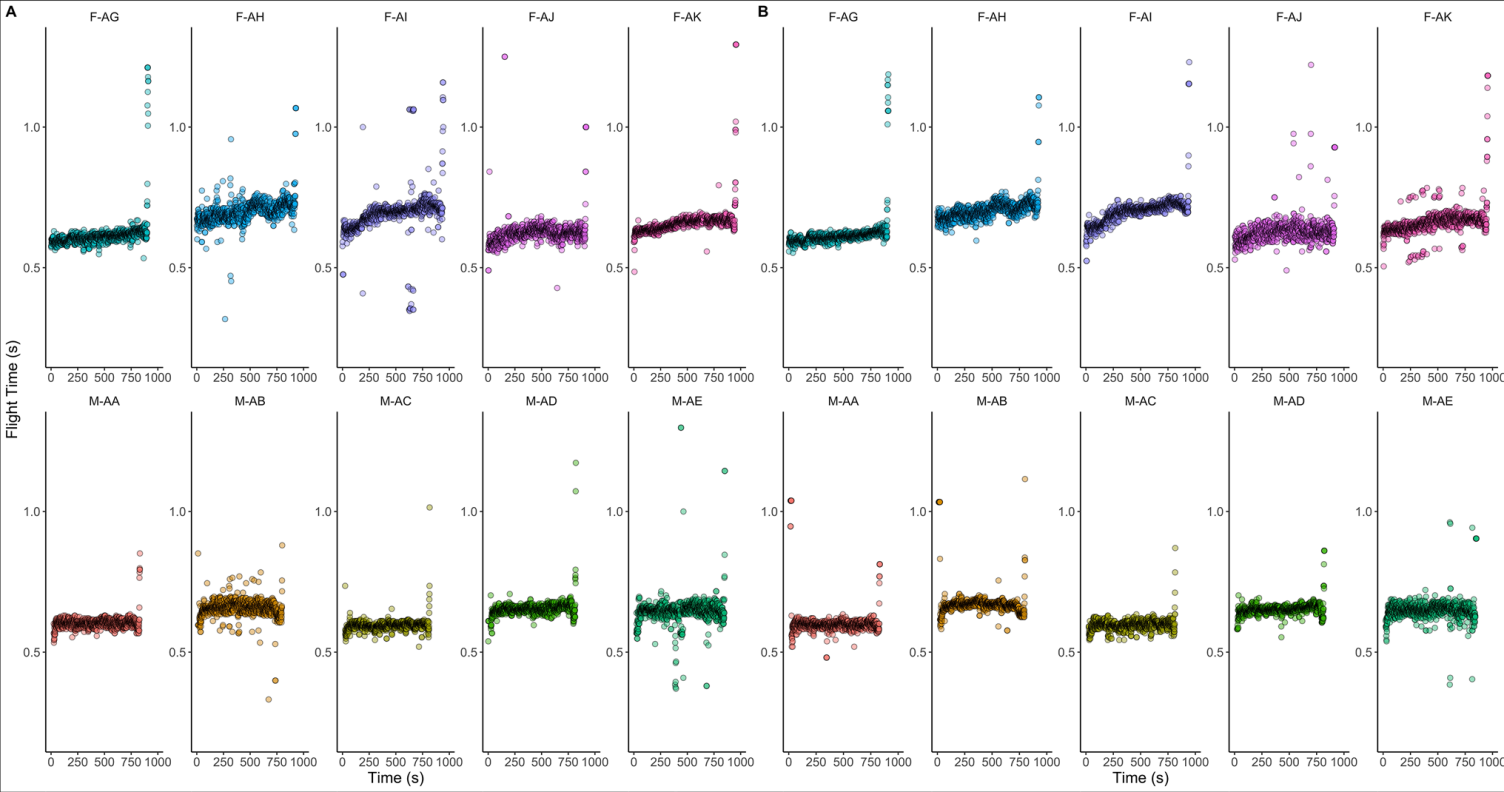
Flight time (ms) of left foot (**a**) and right foot (**b**) during the 5 km event at the 2022 adidas Road to Records event. F: female participants; M: male participants

*Flight Time.* The flight time of individual athletes during the 5 km event varied from 0.59 ± 0.02 s to 0.70 ± 0.04 s in the left foot and from 0.60 ± 0.05 s to 0.70 ± 0.05 s in the right (Fig. 5), with the female athletes showing longer mean flight times (0.65 ± 0.06 s in both feet) compared with males (0.64 ± 0.05 and 0.64 ± 0.04 s).

During the 10 km race, flight time varied from 0.61 ± 0.03 to 0.80 ± 0.25 s in the right foot and from 0.61 ± 0.04 to 0.75 ± 0.21 s in the left, with the female athletes showing longer mean flight times (0.66 ± 0.12 and 0.66 ± 0.10 s, respectively) compared with males (10.62 ± 0.05 s in both feet). Finally, during the 21.1 km event, flight time varied from 0.65 ± 0.05 to 0.69 ± 0.02 s in the right leg and from 0.65 ± 0.05 to 0.68 ± 0.01 s in the left, with female (0.66 ± 0.04 and 0.67 ± 0.04, respectively) and male (0.66 ± 0.03 and 0.66 ± 0.04, respectively) athletes showing similar flight times.

**Fig. 6:**
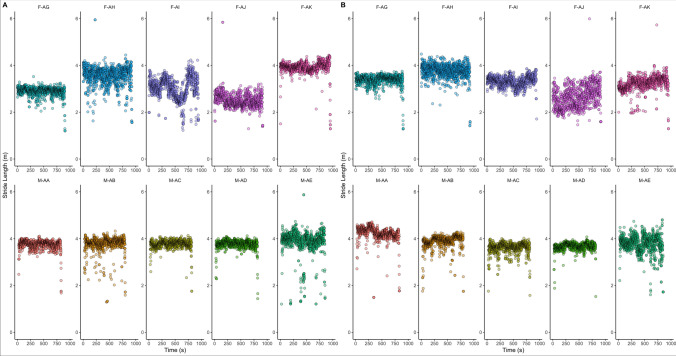
Stride Length (m) of left foot (**a**) and right foot (**b**) during the 5 km event at the 2022 adidas Road to Records event. F: female participants; M: male participants

*Stride Length.* During the 5 km event stride length varied from 2.50 ± 0.30 to 3.92 ± 0.28 m for the left foot, and from 2.65 ± 0.46 to 4.22 ± 0.32 m for the right foot (Fig. 6), with females showing a shorter stride length in both the right (3.26 ± 0.52 m) and the left (3.22 ± 0.63 m) legs compared with males (3.89 ± 0.36 m and 3.78 ± 0.36 m, respectively).

During the 10 km event stride length varied from 2.97 ± 0.80 to 4.21 ± 0.35 m for the left foot, and from 2.76 ± 0.77 to 3.86 ± 0.40 m for the right foot, with females showing a shorter stride length in the right (3.44 ± 0.46 m) but not the left foot (3.64 ± 0.61 m) compared with males (3.50 ± 0.49 and 3.57 ± 0.66 m, respectively). Finally, during the 21.1 km event, stride length varied from 3.23 ± 0.35 to 4.30 ± 0.51 m for the left foot, and from 2.55 ± 0.16 to 4.24 ± 0.21 m for the right foot, with females showing a shorter stride length in both the right (2.95 ± 0.42 m) and the left (3.54 ± 0.34 m) legs compared with males (4.06 ± 0.34 and 3.93 ± 0.49 m, respectively).

### adidas Road to Records: *Discussion*

**Fig. 7:**
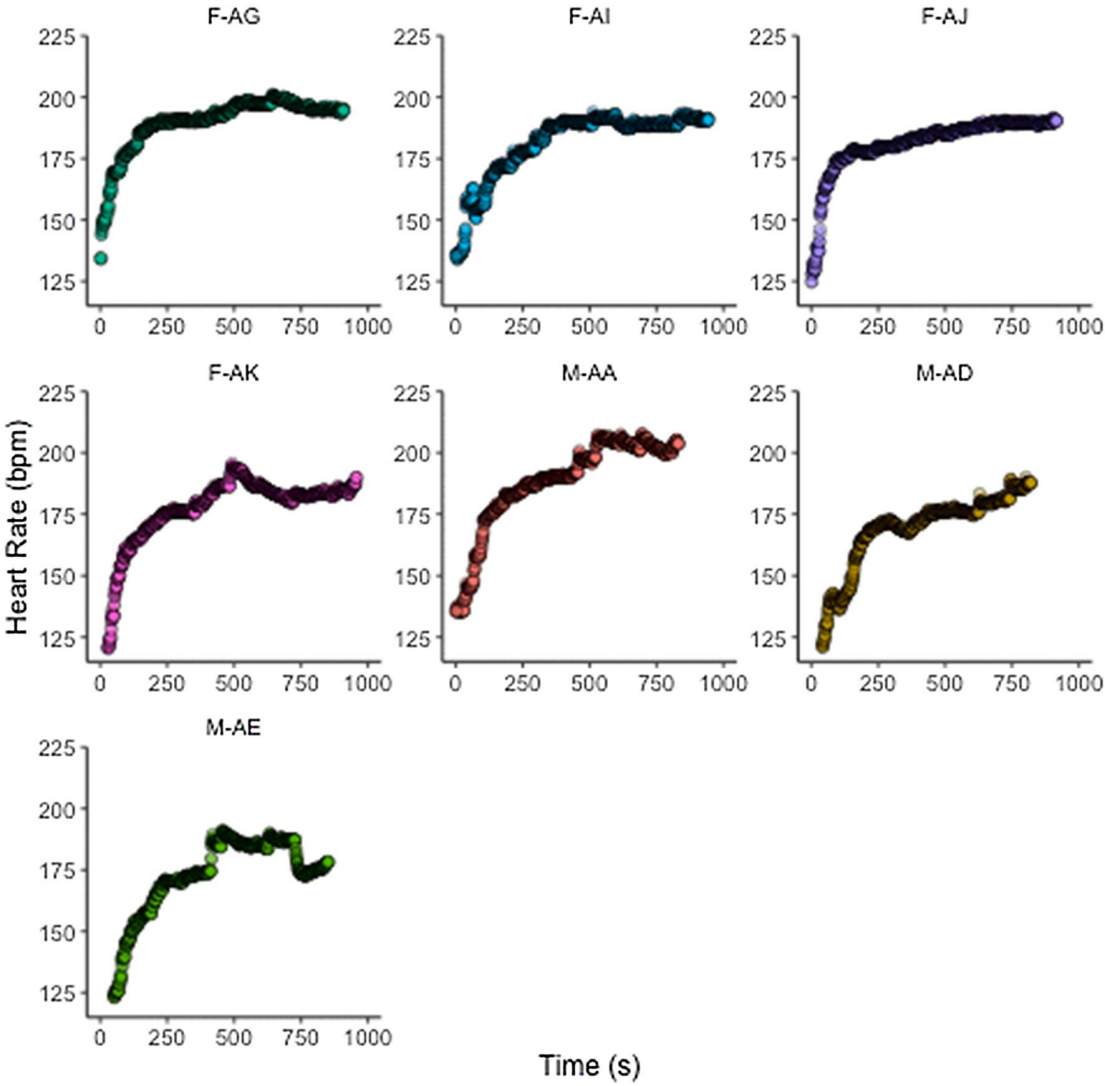
Heart rate (bpm) response to 5 km event during the 2022 adidas Road to Records event. F: female participants; M: male participants


In addition to the capture of data for post-event analysis, allowing for athletes and their support teams to reflect on their performance using objective measures, the major advantage of this data capture approach is that is allows for real-time monitoring of athletes during their event. So, for example, as shown in Fig. 7 (online version), the individual athlete's data can be captured and viewed second by second to allow for performance to be more closely monitored.

(MP4 260 kb)

Although the data that we have collected here are powerful for these individual athlete’s own focus on performance, the real-time data monitoring approach we have developed is more important for the development of elite sport in terms of the incorporation of wearable technologies to ensure the safety of athletes. As set out in the IOC consensus on sporting events in the heat [9], the use of technology to monitor athletes physiological and biomechanical performance indicators in real time during the race is needed to develop effective future strategies for the prevention of injury and illness. In this case study of adidas Road to Records, alongside work that has taken place at Tokyo 2020, we show that athletes can well tolerate this technology and that we can effectively monitor a wide variety of variables allowing for new guardrails for athlete safety to be incorporated in the future.

Our research group has recently shown how this technological approach can be used to monitor core temperature in amateur runners competing in a marathon in temperate conditions, showing that despite the ambient conditions being favourable, a considerable number of runners reached a high core temperature [44]. This approach to monitoring core temperature in real time, alongside new developments in skin temperature sensors to gauge the relationship between core temperature and skin temperature, will be needed at both the Paris 2024 and LA 2028 Olympics due to the high likelihood that these events will be conducted during periods of extreme heat. With the support of the IOC, adidas and our technology partners, we are developing this innovative ecosystem that provides live feedback on land and air temperature, heart rate, core temperature, sweat composition and the biomechanical parameters that we report in this manuscript. All of this is facilitated through a Cloud-based portal allowing the athlete support/medical team to view the data on a desktop, tablet or smartphone in real time anywhere with internet access.

Another key aspect of this development is the ability of members of the public to engage with these data and gain further insight into the performance of elite athletes. There is a drive amongst fans to better understand elite sports performance, and the large amount of data that is generated in elite sport has started to make its way into the psyche of the spectator. A good example of this is the inclusion of strokes gained in golf [45] which has led to a dramatic change in the strategic decisions being made by golfers of all levels. The evolution of the sporting event will include how information on the event is made available to not only athletes, coaches and medical staff but also to the spectator. This is well illustrated in the Tour de France’s partnership with Amaury Sports Organisation to connect fans to the heart of the action during both the Tour de France and the Tour de Femmes [46]. The implementation of this technology and its accessibility to the public or other teams also introduces new issues that should be considered. The performance/physiological data of the athletes will have to be carefully filtered and encrypted so that these cannot be used by other teams to take an advantage over an opponent. Additionally, the sensors/technology allowed to be used during competition, if providing a competitive advantage, will have to be accessible to all competitors to allow for fairness and equity during competition. These and other issues were discussed further by our team in a recent editorial [47].

This technological approach also has telemedicine benefits beyond elite sport, with hospitals, community healthcare providers and patients all potential beneficiaries of this technology. As wearable technology improves, the ability to monitor almost any biological variable remotely, in real time, will revolutionise our ability to treat disease and manage health. This means that healthcare teams will be able to monitor a patient’s key indicators from anywhere in the hospital if they are an in-patient, but also remotely for out-patients, allowing for more effective monitoring and follow-up of patients post treatment.

However, with these exciting developments come challenges, as for this small sample of individuals we have eleven performance variables, with between 1000 (5 km) and 5000 (21 km) data points per athlete. This means that infrastructures, both physical and technical, are needed to be put in place to allow for the appropriate utilisation of this large amount of data. Technical skills amongst athlete support teams, and any potential future users of these approaches, need to be put in place to ensure that the data are not simply collected but utilised. Data science approaches are needed in future practitioners in elite sport to encourage them to fully utilise this revolution in wearable technology for performance analytics.

## Future Recommendations and Directions

The ecosystem developed allows for the remote activation or de-activation of smart devices that collate the information from activated sensors. In previously conducted trials during major city marathons, it was observed that athletes would fail to activate, or in error de-activate the devices under the stress of the event, resulting in lost or uncollected data. This new researcher- or support staff-based approach, alongside the development of a multi-athlete dashboard, allowing many athletes to be monitored simultaneously, represents an exciting development in the monitoring of athletes during competition. This remote monitoring is envisaged to provide useful information for supervising physicians who will be able to access live video feeds alongside the performance and biometrics of individual athletes to inform them of any clinical assessments that may be required.

Future technological innovations will allow for new sensors to be added to the dashboard, meaning that a variety of new biological variables will be able to be measured remotely and in real time. A pertinent example is the non-invasive, in situ monitoring of sweating rate and sweat electrolyte losses via a skin-interfaced wearable microfluidic device with a connected smartphone image processing platform that could soon enable real-time personalised fluid–electrolyte intake recommendations [48]. Another example are devices that incorporate Global Navigation Satellite Systems, accelerometery and gyroscope technology, which are now routinely worn and allow player movement to be recorded and reported live during match play in rugby union [49], as well as within a variety of other contexts with this technology in place for more than two decades and having undergone extensive reliability and validity testing [50]. Local positioning systems are also widely used by indoor sports, having been shown to be valid systems to measure locomotion and positioning [51]. These provide team coaches with key performance metrics such as total distance covered by a player in match play, number of accelerations and decelerations, and impact during any given contact or tackle, with new technologies requiring similar levels of independent assessment to ascertain their efficacy and reliability. Integrating these data, alongside new advances such as smart mouthguards, means that player safety in concussion management becomes more sophisticated. The implementation of instrumented mouthguards during competition has been used to examine head kinematics and assess brain deformation in sports such as football [51]. The correct use and selection of instrumented mouthguards is key, and a recent validity and feasibility study showed how to optimise the adoption of these wearable sensors in sport [52]

As a result of the developments in wearable technology, sporting rules and regulations may need to be altered to facilitate the use of some wearable devices. Some international federations promote the use of wearables in elite sport and, in doing so, encourage companies to develop these tools to facilitate high-level performance. For example, the Technical Rule 6.4.4 of the World Athletics Federations (2022) on ‘assistance to athletes’ allows ‘Heart rate or speed distance monitors or stride sensors or similar devices carried or worn personally by athletes during an event, provided that such device cannot be used to communicate with any other person’. However, Union Cycliste Internationale (UCI) regulations on ‘Onboard Technology’ (Chapter 3: Equipment) state that ‘Devices which capture other physiological data, including any metabolic values such as but not limited to glucose or lactate are not authorized in competition’. Therefore, some reflection and re-evaluation of regulations will be required to ensure that wearable technology is best utilised to ensure that, where appropriate, athlete performance can be monitored to ensure the safety of the athlete, as well as to enhance individual performance and the spectator experience.

Despite the undoubted benefits of such wearables, there are well-founded concerns regarding their use including: the lack of scientific peer-reviewed papers quantifying the potential beneficial effects of analysing specific parameters in each context or in isolation, the quality of hardware and provided data, information overload, data security and exaggerated marketing claims. Therefore, prior to any integration within elite sporting competitions as standard for all participants, several key aspects need to be considered. Firstly, a real benefit to the participants needs to be established. This may be for a performance improvement for the athletes through better understanding of how they can optimise strategy, as well as health monitoring to prevent the high risk of conditions such as exertional heat illness. This benefit may also be via improved engagement of spectators with events, as data are becoming increasingly important within all aspects of spectators' understanding of how events are unfolding through examples such as xG in football, or the stresses and strains of a Formula 1 driver.

Secondly, the hardware itself needs to be established as safe, reliable and valid, as appraised by high-quality independent research. This means that manufacturers and developers should have to substantiate marketing claims of wearable technology with independent scientific evidence so that a global standard of wearable technology can be fully developed [14]. One challenge with this aspect is that the algorithms that are used to determine these variables are the intellectual property of the companies developing these technologies, so establishing the robustness of the science in creating them will become a key aspect of this validation process.

Alongside these discussions, there also needs to be a wider framework for how this technology can be included within elite sporting events, especially with real-time monitoring, which in our opinion is the most exciting element of the work we describe here. The sensors we are using are not novel when used in isolation. However, the integrated use of these sensors within the real-time monitoring ecosystem is unique in competitive sport. Event organisers, including large organisations such as the International Olympic Committee, need to ensure that the infrastructure required for these technologies, including data transmission and security, is included within the planning for these events.

Finally, while the focus of this approach is on using this technology for elite sport, there is undoubted potential for this telemedicine approach within healthcare systems in the future. What we can learn from real-time monitoring of athletes will provide a framework for monitoring of patients both in healthcare settings but also at home with issues such as diabetes, hypertension and falls prevention, through remote monitoring by their healthcare team.

## Conclusions

A new era in precision sport and exercise medicine will include new methods of measuring information in a variety of new and exciting ways. The ability to do this remotely in real time means that we will also need new and more powerful methods ready to integrate with this approach. This focus on technology and implementation during major competition is intended to encourage further innovations enabling future monitoring of a much wider spectrum of data in real time, aiming to understand performance as well as being used as a preventative telemedicine tool to inform the health of athletes during competition and potentially the wider population in the future. The use of such technology, along with other wearable technology transmitting numerous types of data in real time, will undoubtedly become the norm at major sporting events, as international sporting federations seek to make their sport more interesting and accessible to wider audiences.

Wearable technology has the unique capacity to help characterise and understand performance at an individual level, as well as to enhance the broadcast of sporting events with the relay of interesting performance metrics and biometrics to the spectator. This technology has the capacity to revolutionise sport and exercise science, and provides an excellent platform to understand the impact of wearable sensors on performance, wellness, health and disease.

## Declarations

### Data availability

Given the inherent characteristics of this research, the participants in this study did not agree to publicly share the obtained individual data*.*
